# Vitamin D Receptor (VDR) gene polymorphism and risk of ischemic stroke

**DOI:** 10.1186/1755-8166-7-S1-P33

**Published:** 2014-01-21

**Authors:** Puttachandra Prabhakar, Rita Christopher, Dindagur Nagaraja

**Affiliations:** 1 Department of Neurochemistry, National Institute of Mental Health and Neuro Sciences, Bangalore-29, India; 2 Department of Neurology, National Institute of Mental Health and Neuro Sciences, Bangalore-29, India

**Keywords:** VDR gene variants, stroke, multiplex PCR

## Background

Vitamin D deficiency is associated with various chronic disease conditions. The expression and nuclear activation of vitamin D receptor (VDR) are essential for the effect of Vitamin D, because vitamin D is involved in various signaling cascades. Few polymorphisms in VDR gene have been reported to be associated with cardiovascular disease and hypertension. We sought to examine the association between VDR gene variants and risk of ischemic stroke in Indian population.

## Methods

We recruited 250 patients diagnosed with ischemic stroke and 300 age and gender-matched healthy control subjects, after informed consent. 4 SNPs in VDR gene (ApaI, BsmI, FokI & TaqI), were genotyped by using PCR-RFLP method. Serum Vitamin D levels were determined by ELISA. The association of each SNP with the risk of stroke was analyzed by multiple logistic regressions by adjusting with age, gender, smoking, alcohol, hypertension and diabetes.

## Results

The VDR BsmI bb and FokI Ff genotypes were associated with increased risk for ischemic stroke (OR: 1.76; 95% CI; 1.01 – 3.08; P = 0.04) & (OR: 1.52; 95% CI; 0.99 – 2.31; P = 0.05) respectively. No association was observed between TaqI and ApaI polymorphisms and ischemic stroke. Vitamin D deficient (<20ng/ml) subjects with Bsm I Bb & bb genotypes had significantly higher risk for ischemic stroke (OR: 2.24; 95% CI; 1.11 – 4.56; P = 0.03) & (OR: 2.27; 95% CI; 1.05 – 4.99; P = 0.034), respectively. Similarly, increased risk for stroke was observed in Vitamin D deficient subjects with TaqI Tt genotype (OR: 2.77; 95% CI; 1.45 – 5.31; P = 0.002), and ApaI Aa genotype (OR: 2.456; 95% CI; 1.25 – 4.81; P = 0.009).

## Conclusions

In this first study to elucidate the role of VDR gene polymorphism in ischemic stroke patients, we found that genetic variants in VDR gene was associated with an increased risk for stroke especially in Vitamin D deficient subjects. Our findings could contribute to the development of strategies for the prevention of ischemic stroke.

